# A close examination of double filtering with fold change and t test in microarray analysis

**DOI:** 10.1186/1471-2105-10-402

**Published:** 2009-12-08

**Authors:** Song Zhang, Jing Cao

**Affiliations:** 1 Department of Clinical Sciences, University of Texas Southwestern Medical Center, Dallas, Texas, USA; 2 Department of Statistical Science, Southern Methodist University, Dallas, Texas, USA

## Abstract

**Background:**

Many researchers use the double filtering procedure with fold change and *t *test to identify differentially expressed genes, in the hope that the double filtering will provide extra confidence in the results. Due to its simplicity, the double filtering procedure has been popular with applied researchers despite the development of more sophisticated methods.

**Results:**

This paper, for the first time to our knowledge, provides theoretical insight on the drawback of the double filtering procedure. We show that fold change assumes all genes to have a common variance while *t *statistic assumes gene-specific variances. The two statistics are based on contradicting assumptions. Under the assumption that gene variances arise from a mixture of a common variance and gene-specific variances, we develop the theoretically most powerful likelihood ratio test statistic. We further demonstrate that the posterior inference based on a Bayesian mixture model and the widely used significance analysis of microarrays (SAM) statistic are better approximations to the likelihood ratio test than the double filtering procedure.

**Conclusion:**

We demonstrate through hypothesis testing theory, simulation studies and real data examples, that well constructed shrinkage testing methods, which can be united under the mixture gene variance assumption, can considerably outperform the double filtering procedure.

## Background

With the development of microarray technologies, researchers now can measure the relative expressions of tens of thousands of genes simultaneously. However, the number of replicates per gene is usually small, far less than the number of genes. Many statistical methods have been developed to identify differentially expressed (DE) genes. The use of fold change is among the first practice. It can be inefficient and erroneous because of the additional uncertainty induced by dividing two intensity values. There are variants of Student's *t *test procedure that conduct a test on each individual gene and then correct for multiple comparisons. The problem is, with a large number of tests and a small number of replicates per gene, the statistics are very unstable. For example, a large *t *statistic might arise because of an extremely small variance, even with a minor difference in the expression.

The disadvantage of fold-change approach and *t *test has been pointed out by a number of authors [1,2]. There are approaches proposed to improve estimation of gene variances by borrowing strength across genes [1,3,4]. Despite the flaw, fold change and *t *test are the most intuitive approaches and they have been applied widely in practice. To control the error rate, many researchers use fold change and *t *test together, hoping that the double filtering will provide extra confidence in the test results. Specifically, a gene is flagged as DE only if the *p*-value from *t *test is smaller than a certain threshold and the fold change is greater than a cutoff value. For example, in [5], 90 genes were found to be DE with two cutoff values (*p*-value < 0.01 and fold change > 1.5). There are numerous examples in the literature that implement the double filtering procedure with fold change and *t *statistic [6-9]. We argue, however, that the double filtering procedure provides higher confidence mainly because it produces a shorter list of selected genes. Given the same number of genes selected, a well constructed shrinkage test can significantly outperform the double filtering method.

Fold change takes the ratio of a gene's average expression levels under two conditions. It is usually calculated as the difference on the *log*_2 _scale. Let *x*_*ij *_be the log-transformed expression measurement of the *i*th gene on the *j*th array under the control (*i *= 1,⋯, *n *and *j *= 1,⋯, *m*_0_), and *y*_*ik *_be the log-transformed expression measurement of the *i*th gene on the *k*th array under the treatment (*k *= 1,⋯*m*_1_). We define  and .

Fold change is computed by

As for the traditional *t *test, it is usually calculated on the *log*_2 _scale to adjust for the skewness in the original gene expression measurements. The *t *statistic is then computed by

where  is the pooled variance of *x*_*ij *_and *y*_*ik*_. Comparing (1) and (2), it is obvious that fold change and *t *statistic are based on two contradicting assumptions. The underlying assumption of fold change is that all genes share a common variance (on the *log*_2 _scale), which is implied by the omission of the variance component in (1). On the other hand, the inclusion of  in (2) suggests that *t *test assumes gene-specific variances. In order for a gene to be flagged as DE, the double filtering procedure would require the gene to have extreme test scores under the common variance assumption as well as under the gene-specific variance assumption. It is analogous to using the intersection of the rejection regions defined by fold change and *t *statistic.

Assuming a common variance for all the genes apparently is an oversimplification. The assumption of gene-specific variances, however, leads to unstable estimates due to limited replicates from each gene. A more realistic assumption might lie in between the two extremes, i.e., modeling gene variances by a mixture of two components, one being a point mass at the common variance, another being a continuous distribution for the gene-specific variances. Under this mixture variance assumption, a DE gene could have a large fold change or a large *t *statistic, but not necessarily both. Taking intersection of the rejection regions flagged by fold change and *t *statistic, as is adopted by the double filtering procedure, might not be the best strategy under the mixture variance assumption.

The goal of the paper is not to propose a new testing procedure in microarray analysis, but to provide insight on the drawback of the widely used double filtering procedure with fold change and *t *test. We present a theoretically most powerful likelihood ratio (LR) test under the mixture variance assumption. We further demonstrate that two shrinkage test statistics, one from the Bayesian model [10] and the other from the significance analysis of microarrays (SAM) test [1], can be united as approximations to the LR test. This association explains why those shrinkage methods can considerably outperform the double filtering procedure. A simulation study and real microarray data analyses are then presented to compare the shrinkage tests and the double filtering procedure.

## Methods

### A Likelihood Ratio Test

For gene *i*, we use *f*_*i *_= *p*_*v*_*f*_*i*1 _+ (1 - *p*_*v*_)*f*_*i*2_, a mixture of two components *f*_*i*1 _and *f*_*i*2_, to denote the density under the null hypothesis that the gene is not DE under two experiment conditions. Density *f*_*i*1 _is defined under the gene-specific variance assumption, *f*_*i*2 _is defined under the common variance assumption, and *p*_*v *_is the mixing probability. Similarly, we use *g*_*i *_= *p*_*v*_*g*_*i*1 _+ (1 - *p*_*v*_)*g*_*i*2 _to denote the density under the alternative hypothesis, with *g*_*i*1 _and *g*_*i*2 _defined in a similar fashion as *f*_*i*1 _and *f*_*i*2_. For example, in the context of testing DE genes, we can assume *f*_*i*1 _= *N *(*μ*_*i*_, ), *f*_*i*2 _= *N*(*μ*_*i*_, ), *g*_*i*1 _= *N*(*μ*_*i *_+ Δ_*i*_, ), and *g*_*i*2 _= *N*(*μ*_*i *_+ Δ_*i*_, ), where  is the assumed common variance,  is the gene-specific variance, *μ*_*i *_is the mean expression level under the control, and Δ_*i *_is the difference in the expression levels between two conditions. Under the null hypothesis *H*_0 _: Δ_*i *_= 0, the likelihood ratio test statistic, which is the most powerful among all test statistics, is

The *R*_*i *_statistic is a weighted sum of two ratios *g*_*i*1_/*f*_*i*1 _and *g*_*i*2_/*f*_*i*2_, with weight *w*_*i *_= *p*_*v*_*f*_*i*1_/[*p*_*v*_*f*_*i*1 _+ (1 - *p*_*v*_)*f*_*i*2_]. Under the normality assumption, it is easy to show that *R*_*i *_= *w*_*i*_*h*_1_(|*t*_*i*_|) + (1 - *w*_*i*_)*h*_2_(|*fc*_*i*_|), where *fc*_*i *_and *t*_*i *_are fold change and *t *statistic, as defined in (1) and (2). Both *h*_1_(·) and *h*_2_(·) are monotonic increasing functions.

The rejection region of the LR test is defined by *R*_*i *_>*λ*_*R*_, where *λ*_*R *_is the threshold to attain a certain test size. In order to reject *H*_0_, it requires that either |*fc*_*i*_| is large, or |*t*_*i*_| is large, or both. In this sense, the LR test rejection region is more like a union of the rejection regions defined by fold change and *t *statistic. On the other hand, the double filtering procedure with fold change and *t *statistic would require both |*fc*_*i*_| and |*t*_*i*_| to be large. This practice is analogous to using the intersection of the two rejections regions determined by |*fc*_*i*_| and |*t*_*i*_|. Compared with the LR test, the double filtering procedure will lose power. The "loss of power" has two meanings. First, for a given false discovery rate (FDR), the double filtering procedure produces a shorter list of identified genes for further investigation. Second, for a given number of identified genes, the list produced by the double filtering procedure has a higher FDR. The double filtering procedure offers a false sense of confidence by producing a shorter list.

The LR test statistic *R*_*i *_requires one to know the true values of parameters *p*, *μ*_*i*_, , , and Δ_*i*_, which are usually unknown in reality. One strategy is to estimate *R*_*i *_by , where the maximum likelihood estimates (MLE) of the unknown parameters are plugged into (3). Unfortunately, with a small number of replicates from each gene, the MLE would be extremely unstable and lead to unsatisfactory testing results.

A Bayesian model [10] was constructed under the mixture variance assumption to detect DE genes. The inference is made based on the marginal posterior probability of a gene being DE, denoted by *z*_*i *_= *P*(Δ_*i *_≠ 0 | *X, Y*). Here *X *= {*x*_*ij*_} and *Y *= {*y*_*ik*_} are the collection of gene expression data under the two conditions. We will show that, like , *z*_*i *_is also an approximation to *R*_*i*_. The difference between  and *z*_*i *_is that the former plugs in the point estimates (MLE) of unknown parameters, while the latter marginalizes the unknown parameters with respect to their posterior distribution. In the Bayesian inference, the uncertainty from various sources are accounted for in a probabilistic fashion.

Similar to the Bayesian mixture model, some existing methods also try to strike a balance between the two extreme assumptions of a common variance and gene-specific variances. The SAM statistic slightly modifies the *t*-statistic by adding a constant to the estimated gene-specific standard deviation in the denominator. We will present it as being motivated by a mixture model on the variances (standard deviations). Furthermore, the SAM statistic can be directly written as a weighted sum of *t *statistic and fold change. Thus both the Bayesian method and the SAM method are approximations to the LR test under the mixture variance assumption, and they can achieve better performance than the double filtering procedure.

### The Bayesian Mixture Model

Cao *et al*. [10] proposed a Bayesian mixture model to identify DE genes, which has shown comparable performance to frequentist shrinkage methods [1,11]. With parameters (*μ*_*i*_, Δ_*i*_, , , *p*_*v*_) defined similarly as in the LR test, gene expression measurements *x*_*ij *_and *y*_*ij *_are modeled by normal distributions with a mixture structure on the variances,

A latent variable *r*_*i *_is used to model the expression status of the *i*th gene,,

where *r*_*i *_= 0/1 indicates that gene *i *is non-DE/DE and it is modeled by a Bernoulli distribution: *r*_*i *_| *p*_*r *_~ *Bernoulli*(*p*_*r*_). For  and , it is assumed that  and  ~ IG(*a*_0_, *b*_0_). Here IG(*a, b*) denotes an inverse gamma distribution with mean *b*/(*a *- 1). The other hyper-priors include, *μ*_*i *_~ *N*(0, ), *p*_*r *_~ *U*(0, 1), and *p*_*v *_~ *U*(0, 1). More details can be found in [10].

We make inference based on *z*_*i *_= *P*(*r*_*i *_= 1 | *X, Y*) = *P*(Δ_*i *_≠ 0 | *X, Y*), the marginal posterior probability that gene *i *is DE. A gene is flagged as DE if *z*_*i *_>*λ*_*z*_, where *λ*_*z *_is a certain cutoff. We argue that the Bayesian rejection region defined by *z*_*i *_>*λ*_*z *_is an approximation to the LR test rejection region defined by *R*_*i *_>*λ*_*R*_. First we have

Here *P*(*μ*_*i*_, Δ_*i*_, , , *p*_*v*_, *p*_*r *_| *X, Y*) is the joint posterior distribution of (*μ*_*i*_, Δ_*i*_, , , *p*_*v*_, *p*_*r*_), marginalized with respect to other random parameters (e.g., *μ*_*j *_and , *j *≠ *i*).

It is easy to show that

Given parameters (*μ*_*i*_, Δ_*i*_, , , *p*_*v*_, *p*_*r*_), *P*(*r*_*i *_= 1 | *μ*_*i*_, Δ_*i*_, , , *p*_*v*_, *p*_*r*_, *X, Y*) is an increasing function of *R*_*i*_. Rejecting *H*_0 _for *R*_*i *_>*λ*_*R *_is equivalent to rejecting for *P*(*r*_*i *_= 1 | *λ*_*i*_, Δ_*i*_, , , *p*_*v*_, *p*_*r*_, *X, Y*) >*λ*_*z*_, with *λ*_*z *_= *λ*_*R*_/[*λ*_*R *_+ (1 - *p*_*r*_)/*p*_*r*_]. Thus the two test statistics, *P*(*r*_*i *_= 1 | *μ*_*i*_, Δ_*i*_, , , *p*_*v*_, *p*_*r*_, *X, Y*) and *R*_*i*_, are equivalent. Expression (5) demonstrates that *z*_*i *_is obtained from *P*(*r*_*i *_= 1 | *μ*_*i*_, Δ_*i*_, , , *p*_*v*_, *p*_*r*_, *X, Y*) by integrating with respect to the unknown parameters under the joint posterior distribution. If the integral does not have a closed form, we can conduct numerical integration to calculate *z*_*i *_through the Gibbs sampling algorithm [12,13]. The uncertainty from those unknown parameters are accounted for in a probabilistic fashion. It is in this sense that we consider *z*_*i *_a good approximation to the LR test statistic *R*_*i*_.

### The SAM Test

The SAM statistic [1] is defined as

where *s*_*i *_is the gene-specific standard deviation, and *s*_0 _is a constant that minimizes the coefficient of variation. Although it might not be the original intention of the authors [1], a test statistic like *d*_*i *_can be motivated by a model with a mixture structure on gene standard deviations. We begin with a simple case where *x*_*ij *_~ *N*(*μ*_*i*_, ) and *y*_*ik *_~ *N*(*μ*_*i *_+ Δ_*i*_, ), and the null hypothesis is *H*_0 _: Δ_*i *_= 0. Given *δ*_*i*_, the LR test statistic is

We assume a mixture structure on gene standard deviations, where *δ*_*i *_= *σ*_*i *_with probability *p*_*v *_and *δ*_*i *_= *σ*_0 _with probability 1 - *p*_*v*_. We can then approximate  by

Replacing *σ*_*i *_with *s*_*i *_and  with *s*_0_, we can see that *d*_*i *_and  only differ by a factor of 1/*p*, which does not change the ordering of test statistics. The above derivation suggests that the SAM statistic can also be considered an approximation to the LR test statistic under the mixture variance (standard deviation) assumption. We can also write *d*_*i *_as a weighted sum of *t*_*i *_and *fc*_*i*_:

Recall that under the mixture variance assumption, the LR test statistic is *R*_*i *_= *w*_*i*_*h*_1_(|*fc*_*i*_|) + (1 - *w*_*i*_)*h*_2_(|*t*_*i*_|), where *h*_1_(·) and *h*_2_(·) are both monotonic increasing functions. Both *d*_*i *_and *R*_*i *_define rejection regions that are analogous to the union of the rejection regions defined by *t *test and fold change. In other words, the SAM procedure rejects *H*_0 _for large |*t*_*i*_|, or large |*fc*_*i*_|, or both. The SAM statistic is a better approximation to the LR test statistic than the double filtering procedure.

As a side note, Cui *et al*. [11] proposed a shrunken *t *test procedure based on a variance estimator that borrow information across genes using the James-Stein-Lindley shrinkage concept. This variance estimator shrinks individual variances toward a common value, which conceptually serves the same purpose as the mixture variance model. From this perspective, we also consider the shrunken *t *statistic an approximation to the LR test statistic.

## Results and Discussion

### Simulation Study

We conducted a simulation study to compare the double filtering procedure to the shrinkage methods. The simulation truth is specified as follows. We tested 1000 genes with 100 genes being truly DE. Without loss of generality, we set *μ*_*i *_= 0. We further assumed

and

Three scenarios were considered. Scenario 1: 90% of the genes with gene-specific variances and 10% of the genes with a common variance, and 3 replicates per gene under each condition. Scenario 2: same as Scenario 1, but with 6 replicates per gene under each condition. Scenario 3: all the genes having a gene-specific variance, and 3 replicates per gene under each condition. For each scenario we repeated the simulation 1000 times.

For the Bayesian mixture model, we specified noninformative priors so that the posterior inference is dominated by information from data. We let  =  = 5.0 where 5.0 is sufficiently large for expression levels on the logarithm scale. To specify the hyper-parameters for the inverse gamma priors, first we set *a*_*σ *_= *a*_0 _= 2.0 so that the inverse gamma priors have an infinite variance. Then we set the prior means,  and , equal to the average of the sample variances to solve for *b*_*σ *_and *b*_0_. Finally, we chose *a*_*r *_= *b*_*r *_= *a*_*v *_= *b*_*v *_= 1, which corresponds to uniform priors for *p*_*r *_and *p*_*v*_.

**Figure 1:**
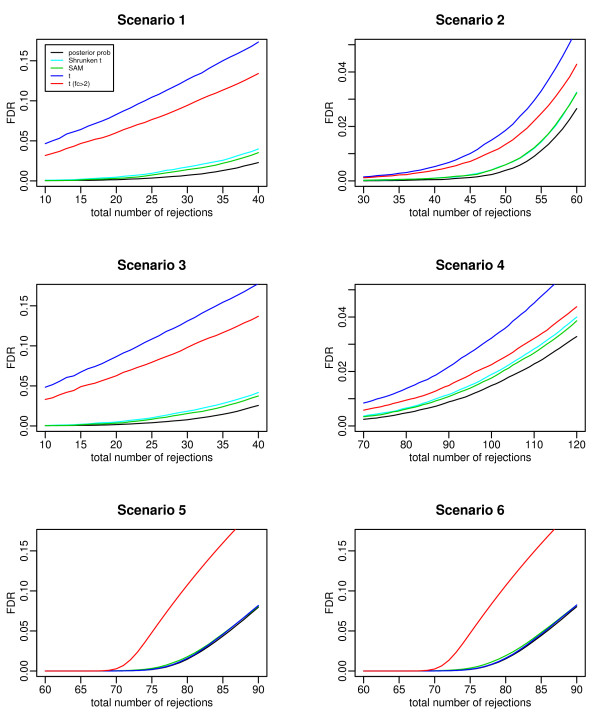
**Comparison of the FDR given the total number of selected genes under Scenario 1-6 in the simulation study**. The competing test statistics are the posterior probability based on the Bayesian model, the shrunken *t *statistic, the SAM statistic, the *t *statistic, and the double filtering procedure with *t *statistic and fold change.

Five test statistics were compared: the marginal posterior probability (*z*_*i*_) of a gene being DE based on the Bayesian mixture model, the SAM statistic, the shrunken *t *statistic, the *t *statistic, and the double filtering with *t *statistic and fold change greater than 2. The first three graphs in Figure 1 plot the FDR versus the total number of selected genes under the above three scenarios. The shrinkage methods (the Bayesian model, the SAM test, and the shrunken *t *test) have comparable performance. The double filtering procedure performs better than the traditional *t *statistic, but it is obviously outperformed by the three shrinkage methods. We have tried different fold change cutoff values for the double filtering procedure (e.g., setting the cutoff at 1.5) and the results did not change materially. Given the same number of selected genes, the shrinkage methods can identify more truly DE genes than the double filtering procedure. Note that under the gene-specific variance assumption (Scenario 3), the *t *test, which theoretically is the most powerful likelihood ratio test, still performs the poorest. This result indicates the usefulness of shrinkage in microarray studies, where only a small number of replicates are available for each gene. In short, the simulation study shows that for a given number of selected genes, well constructed shrinkage methods can outperform the double filtering procedure.

In Scenario 1 and 2 of the simulation study, the true variance distribution is specified as the mixture of a point mass and an inverse gamma distribution, which might lead to a result that is biased in favor of a shrinkage method. Here we conduct another simulation study with a "real" variance distribution, denoted as Scenario 4. Specifically, let *x*_*ij *_(*j *= 1, ... , *m*_0*i*_) and *y*_*ik *_(*k *= 1, ... , *m*_1*i*_) be the observed expression levels from a real microarray study.

Define the residual vector **e_i _**=(*e*_*i*1_, ... , )' by

Then **e_i _**can be considered a set of random errors sampled based on the true variance distribution. We simulate 1000 data sets according to the following steps. For iteration *s *(*s *= 1, ⋯, 1000) and gene *i *(*i *= 1, ... , *n*),

1. obtain a random permutation of (*e*_*i*1_, ... , ), denoted by ;

2. generate  as described in the previous simulation scenarios;

3. for *j *= 1, ... , *m*_0*i*_, compute  = , and for *k *= 1, ... , *m*_1*i*_, compute , where  is the *j*th element of .

The real data comes from a microarray study comparing the gene expressions of breast cancer tumors with *BRCA1 *mutations, *BRCA2 *mutations, and sporadic tumors [14]. The data set is available at http://research.nhgri.nih.gov/microarray/NEJM_Supplement. Here we only consider the *BRCA1 *group and the *BRCA2 *group. There are 3226 genes, with 7 arrays in the *BRCA1 *group and 8 arrays in the *BRCA2 *group. We analyzed the data on the *log*_2 _scale. Following Storey and Tibshirani [15], we eliminated genes with aberrantly large expression values (>20), which left us with measurements on *n *= 3169 genes. The fourth graph in Figure 1 compares the different methods under Scenario 4, where the residual vector **e_i _**was constructed based on the breast cancer data. We kept the same replicate number in the experiment, with 7 replicates per gene in one group and 8 replicates in the other group. The relative performance of the five methods remains unchanged as in the other scenarios.

In current microarray studies, the number of replicates per gene can be easily 30 or more due to the low cost of array and the easiness to collect patients. So we considered two scenarios with a relatively large number of replicates. Scenario 5: 90% of the genes with gene-specific variances and 10% of the genes with a common variance, and 30 replicates per gene under each condition. Scenario 6: all the genes having a gene-specific variance, and 30 replicates per gene under each condition. In each of the two scenarios, we assume there are 1000 genes with 100 genes being truly DE. The two graphs in the bottom panel of Figure 1 plot the FDR versus the total number of selected genes for the five test statistics under Scenario 5 and Scenario 6, respectively. The comparison demonstrates that when the replicate number is large, the performance of the traditional *t *test becomes comparable to the performance of the shrinkage methods, thanks to the more reliable estimate of gene variance component. More importantly, the drawback of the double filtering procedure becomes more obvious, which has substantially worse performance compared to the other methods, including the *t *test.

### Experimental Datasets

**Figure 2:**
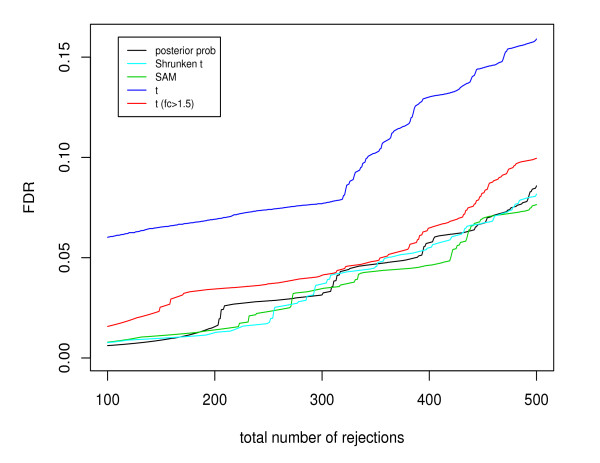
**Comparison of the FDR given the total number of selected genes in the analysis of Golden Spike data**. The test statistics include the posterior probability based on the Bayesian model, the shrunken *t *statistic, the SAM statistic, the *t *statistic, and the double filtering procedure with *t *statistic and fold change.

In this section we compared the shrinkage methods with the double filtering procedure based on two microarray datasets. The first is the Golden Spike data [16] where the identities of truly DE genes are known. The Golden Spike dataset includes two conditions, with 3 replicates per condition. Each array has 14,010 probesets, among which 10,144 have non-spiked-in RNAs, 2,535 have equal concentrations of RNAs, and 1,331 are spiked-in at different fold-change levels, ranging from 1.2 to 4-fold. Compared with other spike datasets, the Golden Spike dataset has a larger number of probsets that are known to be DE, making it popular for comparing performance among different methods. Irizarry *et al*. [17] pointed out that "the feature intensities for genes spiked-in to be at 1:1 ratios behave very differently from the features from non-spiked-in genes". Following Opgen-Rhein and Strimmer [18], we removed the 2,535 probe sets for spike-ins with ratio 1:1 from the original data, leaving in total 11,475 genes and 1,331 known DE genes. Figure 2 plots the FDR under each testing procedures versus the total number of rejections. For the double filtering procedure, the fold change cutoff was set at 1.5 because only 248 genes have a fold change greater than 2.0. The figure indicates that the shrinkage methods (Bayesian, SAM, and shrunken *t*) have similar performance, and they outperform the double filtering procedure and *t *test.

**Figure 3:**
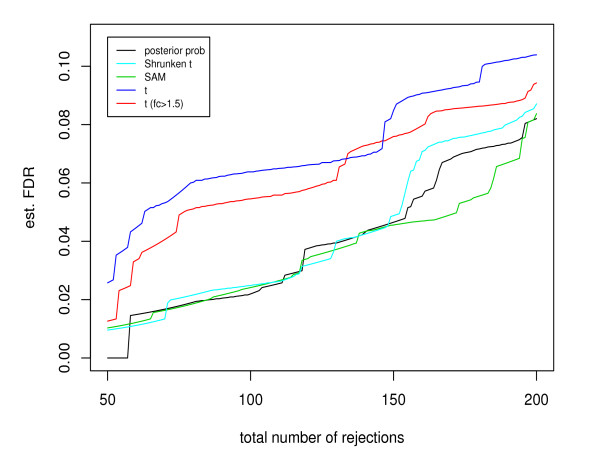
**Comparison of the estimated FDR given the total number of selected genes in the analysis of the breast cancer data**. The test statistics include the posterior probability based on the Bayesian model, the shrunken *t *statistic, the SAM statistic, the *t *statistic, and the double filtering procedure with *t *statistic and fold change.

The second is the breast cancer dataset [14] described in the simulation study. With the identities of truly DE genes unknown, we estimated the FDR for the SAM test, the shrunken *t *test, the *t *test, and the double filtering procedure, through the permutation approach described in [15]. For Bayesian methods, Newton *et al*. [19] proposed to compute the Bayesian FDR, which is the posterior proportion of false positives relative to the total number of rejections. However, the Bayesian FDR is incomparable to the permutation-based FDR estimate employed by frequentist methods [20]. Cao and Zhang [21] developed a generic approach to estimating the FDR for Bayesian methods under the permutation-based framework. A computationally efficient algorithm was developed to approximate the null distribution of the Bayesian test statistic, the posterior probability. The approach can provide an unbiased estimate of the true FDR. Constructed under the same permutation-based framework, the resulting FDR estimate allows a fair comparison between full Bayesian methods with other testing procedures. We adopted the approach in [21] to estimate the FDR of the Bayesian mixture model (4). Figure 3 plots the permutation-based FDR estimates under each testing procedure versus the total number of rejections. It shows that the shrinkage methods can considerably outperform the double filtering procedure.

## Conclusion

It has been a common practice in microarray analysis to use fold change and *t *statistic to double filter DE genes. In this paper, we provided a close examination on the drawback of the double filtering procedure, where fold change and *t *statistic are based on contradicting assumptions. We further demonstrated that several shrinkage methods (SAM, shrunken *t*, and a Bayesian mixture model) can be united under the mixture gene variance assumption. Based on the theoretical derivation, the simulation study, and the real data analysis, we showed compelling evidence that well constructed shrinkage methods can outperform the double filtering procedure in identifying DE genes. With publicly available softwares, these methods are as easy to implement as the double filtering procedure.

We acknowledge some researchers' argument that the double filtering procedure might work well because it filters out the genes that show relatively small differences between conditions, which are sometimes considered to be less biologically meaningful. This argument, however, is based on the criterion of so called "biological meaningfulness" instead of testing power. Although many biologists refer to fold change in terms of "biological meaningfulness", there is in fact no clear cut-off for it, and 2-fold is often invoked merely based on convenience. In addition, different normalization methods can differ quite drastically in terms of the fold changes they produce. So a particular cut-off in fold change could mean one thing using one method and quite another using a different method. Taken together, even if researchers decide to employ the double filtering procedure based on the rationale of "biological meaningfulness", it is still helpful to understand the potential loss in power.

## Authors' contributions

SZ and JC conceived the study, conducted the examination on the double filtering procedure, analyzed the data, and drafted the paper. All authors read and approved the final manuscript.
